# Preserved satellite cell pool in a laminin-α2-deficient model of congenital muscular dystrophy

**DOI:** 10.1242/dmm.052782

**Published:** 2026-09-30

**Authors:** Luiza Albuquerque Coelho, Antônio Fernando Ribeiro Junior, Letícia Nogueira Feitosa, Felipe Tadeu Galante Rocha de Vasconcelos, Job Carvalho Bezerra, Mariz Vainzof

**Affiliations:** Human Genome and Advanced Therapies Center, Institute of Bioscience, University of São Paulo, 05508-090 São Paulo, Brazil

**Keywords:** Laminin-α2, Congenital muscular dystrophy, Degeneration, Regeneration, Satellite cells

## Abstract

Deficiency in the α2 subunit of laminin-211, encoded by *LAMA2*, causes congenital LAMA2-related muscular dystrophy (LAMA2 MD), characterized by severe and progressive muscle weakness. In this study, we analyzed muscle regeneration and degeneration in the gastrocnemius muscle of dy^2J^ mice, a model of LAMA2 MD, across different ages. We found that the proportion of satellite cells (SCs) per fiber was significantly higher in the dy^2J^ groups compared to that in the wild-type (WT) group, and that SC numbers did not decrease with disease progression. The percentages of activated SCs, and the expression of *Myog* and *Tgfb*, were also significantly elevated in affected animals compared to those in WT animals. Despite the preserved SC pool and their activation, the percentage and diameter of newly regenerated fibers progressively declined as the disease advanced. Histopathological analysis further revealed progressive fiber muscle atrophy. These results suggest that the impairment of muscle regeneration with time in the dy^2J^ model is not due to SC depletion but rather a failure at the later stages of SC differentiation. Further investigation into these processes is essential for developing effective treatment strategies for LAMA2 MD.

## Teaser

**Summary:** Progressive impairment of muscle regeneration in the dy^2J^ mouse model of LAMA2-related muscular dystrophy arises from failure at late stages of satellite cell differentiation rather than depletion of the satellite cell pool.

## INTRODUCTION

Muscular dystrophies are a genetically and biochemically heterogeneous group of diseases characterized by progressive and irreversible loss of muscular fibers, leading to muscle weakness and impaired motor function (Carter et al., 2018; Mercuri and Muntoni, 2013). This deterioration results from repeated cycles of muscle degeneration combined with insufficient capacity for muscle repair, ultimately causing the replacement of muscle tissue with fibro-fatty tissue (Bencze, 2022; Wallace and McNally, 2009). Variants in the *LAMA2* gene cause congenital LAMA2-related muscular dystrophy (LAMA2 MD), the most common form of congenital muscular dystrophy. *LAMA2* encodes the α2 chain of the laminin-211 (α2β1γ1) heterotrimer, which is expressed in the skeletal muscle and is essential for basement membrane stability (Yurchenco et al., 2018). Consequently, deficiency in the α2 chain destabilizes the basal lamina, increasing susceptibility to mechanical stress and fiber damage (Topaloğlu and Poorshiri, 2024). To investigate the mechanisms of LAMA2 MD and test potential therapies, several animal models have been used, including the dy^2J^ mouse.

In the dy^2J^ mouse, symptoms first appear at 3 weeks of age and are characterized by hindlimb retraction when the animal is suspended by the tail. Disease progression is marked by muscle weakness, loss of body weight, increased deposition of extracellular matrix (ECM) components and permanent hindlimb paralysis (Gawlik and Durbeej, 2020; Yurchenco et al., 2018). This phenotype results from a point mutation in a donor splicing site of the *Lama2* gene (Kuang et al., 1998; Xu et al., 1994), which causes the deletion of 57 amino acids and a glutamine-to-glutamate substitution in the α2LN domain. Each laminin chain has a LN domain at the N-terminal end, which is critical for laminin self-polymerization; therefore, alterations in these domains prevent proper polymerization (Holmberg and Durbeej, 2013; Sunada et al., 1995). Beyond these structural defects, disease progression in dy^2J^ mice also reflects the interplay between impaired muscle regeneration and increased fibrosis.

The repeated attempts of muscle regeneration depend on the presence and activity of satellite cells (SCs) (Mercuri and Muntoni, 2013). Quiescent SCs express only *Pax7*, and their commitment is driven by the expression of myogenic regulatory factors (MRFs). Specifically, upon activation, myoblast precursor determination depends on *Myf5* and *Myod* (also known as *Myod1*), whereas terminal muscle differentiation requires *Myog*, *Myod* and *Mrf4* (also known as *Myf6*) (Zanou and Gailly, 2013). In contrast, fibro-fatty tissue proliferation is regulated by TGF-β1, a protein that inhibits myoblast differentiation, promotes the conversion of myoblasts into fibroblasts and stimulates the expression of ECM proteins, such as collagen (Delaney et al., 2017).

This study aimed to investigate muscle regeneration and degeneration in the gastrocnemius muscle of dy^2J^ mice across different ages and stages of the disease. Regeneration was studied by the analysis of the proportion of SCs and their activation, expression of *Pax7* and selected MRFs, and the quantification of newly regenerated fibers. Degeneration and muscle loss was measured by histological marks of degeneration and the expression of genes associated with fibrotic tissue replacement.

## RESULTS

### The SC pool is preserved during disease progression in dy^2J^ muscle

Immunofluorescence for PAX7 and KI67 (also known as MKI67) was performed to analyze the proportion of total SCs per muscle fiber and to determine the percentages of these cells that were activated (Fig. 1A). Significantly higher numbers of SCs per fiber were observed in all groups of dy^2J^ mice compared to those in controls, with no significant differences among dy^2J^ groups (Fig. 1B). Similarly, dy^2J^ groups 1, 2 and 4 showed significantly higher percentages of activated SCs than did the wild-type (WT) group, but there were no significant differences in percentages among the dy^2J^ groups (Fig. 1C). However, it is important to note that the *P*-values for differences in percentages of activated SCs between group 1 and the other dy^2J^ groups were 0.052, 0.078 and 0.078 when comparing group 1 to groups 2, 3 and 4, respectively.

**Fig. 1.**
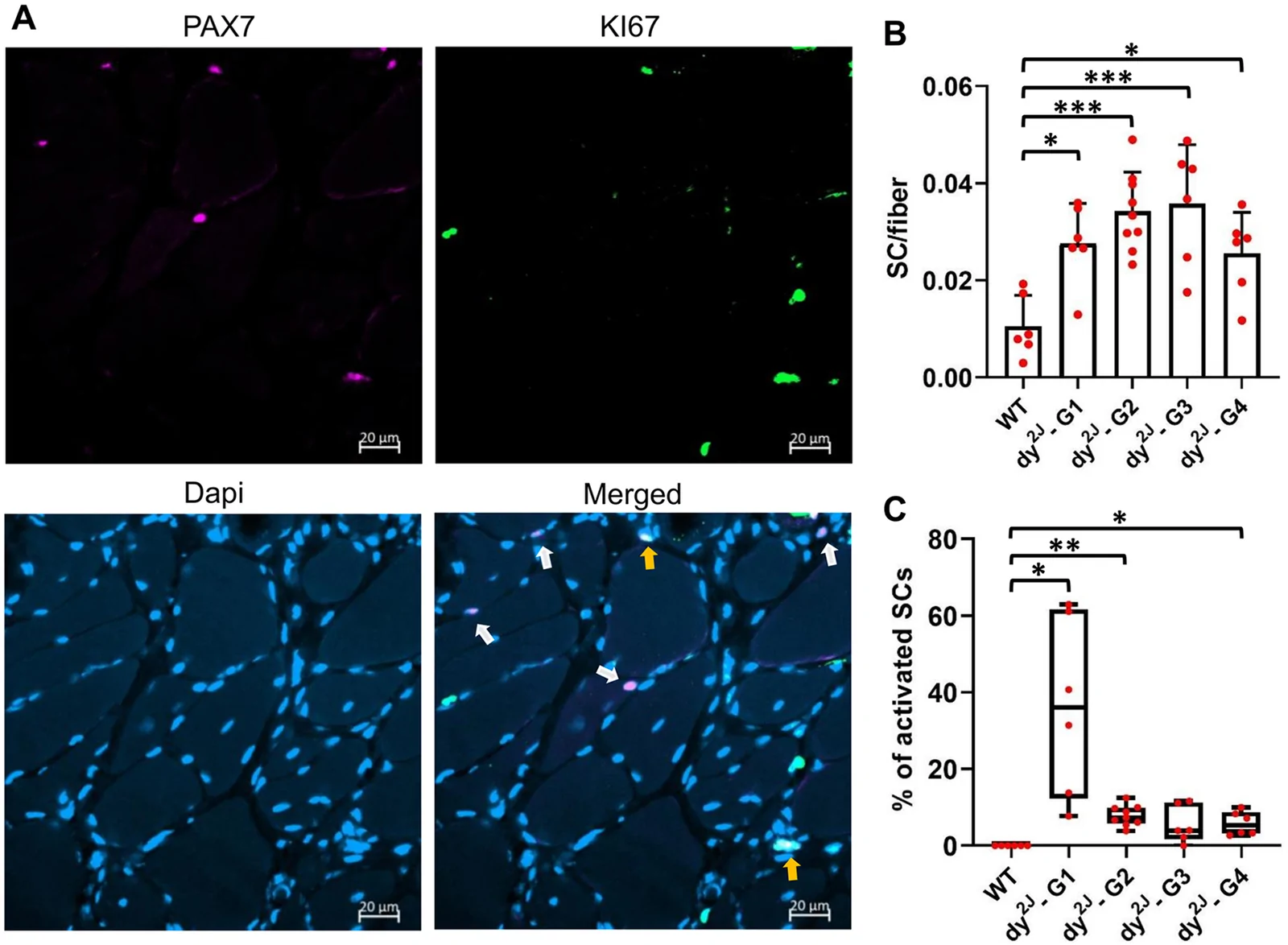
**Quantification of satellite cells (SCs).** (A) Immunofluorescence staining of PAX7 (magenta) and KI67 (green). White arrows indicate quiescent SCs; yellow arrows indicate activated SCs. (B) Proportion of SCs per muscle fiber in the young-adult wild-type (WT) group and in the four dy^2J^ age groups. Data represent mean±s.d. (C) Percentage of activated SCs in the WT group and dy^2J^ age groups. Box-and-whisker plots show median (center line), 25th-75th percentiles (box limits) and minimum-to-maximum range (whiskers). Individual data points represent biological replicates (individual animals). At least 1000 fibers were analyzed per animal. Group (G) sizes: WT, six animals; dy^2J^-G1, seven animals; dy^2J^-G2, nine animals; dy^2J^-G3, six animals; dy^2J^-G4, six animals. Statistical analysis was performed using one-way ANOVA for SCs/fiber (*P*<0.001), with Tukey post hoc for multiple comparisons. For the percentage of activated SCs, Kruskal-Wallis (*P*<0.001) test was performed, with Dwass–Steel–Critchlow–Fligner method (DSCF) for multiple comparisons. *P<0.05, **P<0.01 and ***P<0.001.

### Myogenic factor expression is maintained over time in dy^2J^ laminin-α2-deficient muscle

Investigating the expression of myogenic factors in dystrophic mice is essential for understanding the differentiation patterns of SCs. To clarify how SC differentiation evolves with disease progression, we performed quantitative PCR (qPCR) analysis of *Pax7*, *Myf5*, *Myod* and *Myog*, using the *Tbp* gene as endogenous, in the four age groups of dy^2J^ mice and in a group of 3-month-old WT mice. We observed no significant differences in the expression of *Pax7*, *Myf5* and *Myod* between the WT group and any of the dy^2J^ age groups (Fig. 2A-C). In contrast, *Myog* expression was significantly higher in dy^2J^ groups 1, 2 and 4 than in the WT group. Furthermore, group 4 had significantly lower relative expression of *Myog* than group 1 (Fig. 2D).

**Fig. 2.**
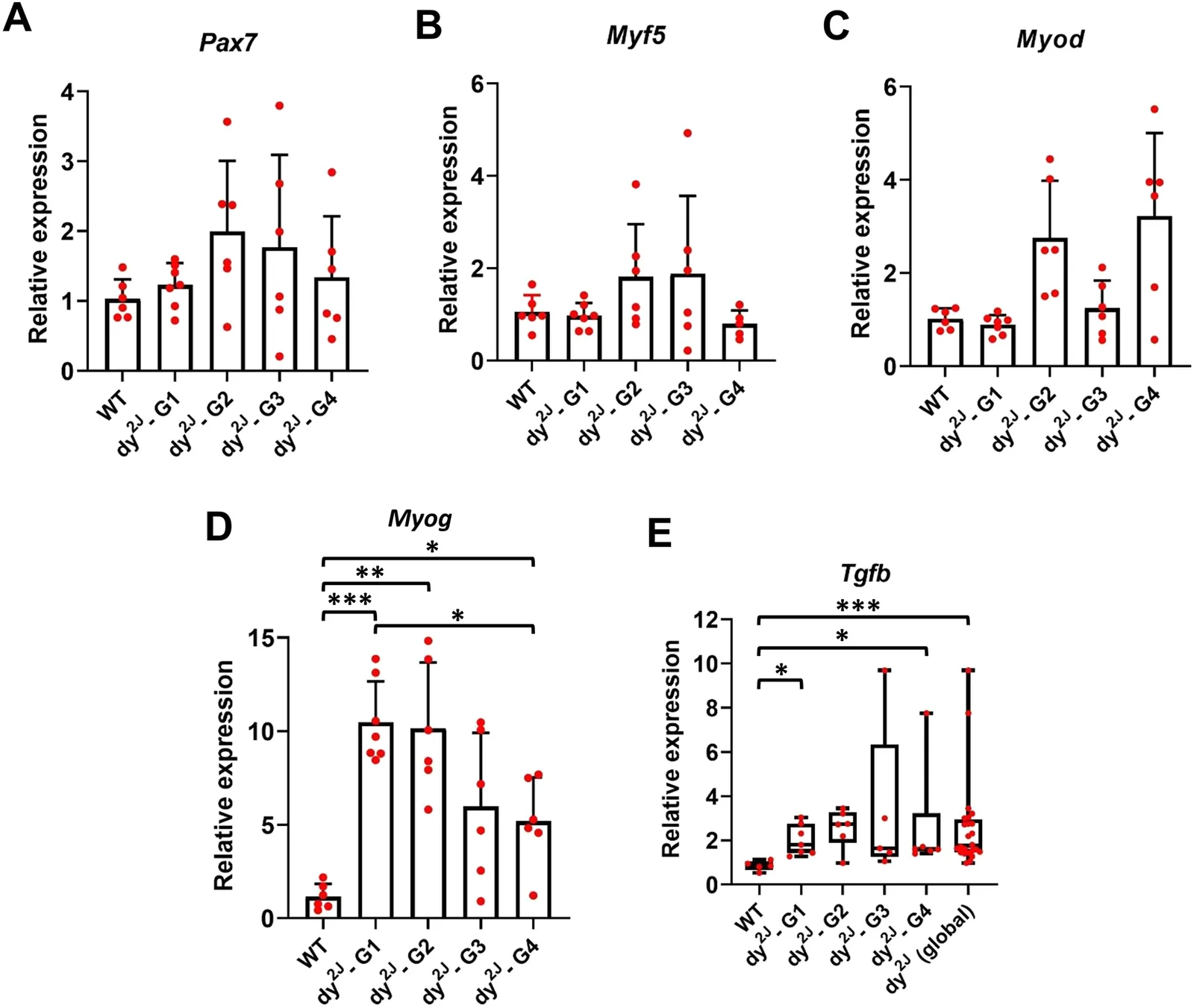
**Expression of regeneration- and degeneration-related genes in the different dy^2J^ age groups and 3-month-old WT group.** (A-D) Relative expression of *Pax7* (A), *Myf5* (B), *Myod* (C) and *Myog* (D). Bars represent mean±s.d. Group sizes: WT, six animals; dy^2J^-G1, seven animals; dy^2J^-G2, six animals; dy^2J^-G3, six animals; dy^2J^-G4, six animals. Statistical analysis was performed using one-way ANOVA (*P*=0.253 for *Pax7*, *P*=0.445 for *Myf5*, *P*=0.012 for *Myod* and *P*<0.001 for *Myog*), with Games–Howell post hoc for multiple comparisons. (E) Relative expression of *Tgfb*. Box-and-whisker plots show median (center line), 25th-75th percentiles (box limits) and minimum-to-maximum range (whiskers). Group sizes: WT, five animals; dy^2J^-G1, seven animals; dy^2J^-G2, six animals; dy^2J^-G3, five animals; dy^2J^-G4, six animals. Statistical analysis was performed using Kruskal–Wallis test, with DSCF for multiple comparisons. Relative gene expression was calculated using the 2^−ΔΔCT^ method, normalized to *Tbp* and expressed relative to that in the 3-month-old WT control group. Individual points represent independent biological replicates (individual animals), and quantitative PCR reactions were run in technical triplicates. *P<0.05, **P<0.01 and ***P<0.001.

To further investigate factors associated with muscle degeneration during disease progression, qPCR was used to analyze the expression of *Tgfb* (also known as *Tgfb1*). The collective dy^2J^ groups exhibited significantly higher relative *Tgfb* expression than the WT group, a finding also confirmed when comparing group 1 and group 4 individually to the WT control (Fig. 2E).

### Newly regenerated fibers are smaller and more frequent in dy^2J^ muscles than in WT muscles, and their abundance decreases with disease progression

To investigate the regenerative process with disease progression in the dy^2J^ lineage, we used immunofluorescence to analyze the frequency and diameter of newly regenerated fibers, identified by the presence of developmental myosin heavy chain (dMyHC), across the four dy^2J^ age groups and in the WT group (Fig. 3A). The gastrocnemius of an uninjured adult mouse typically does not present dMyHC^+^ fibers (Schiaffino et al., 2015), as was observed in WT samples. In contrast, all dy^2J^ age groups had newly regenerated fibers, but their frequency declined as disease progressed (Fig. 3B). Additionally, group 1 presented larger dMyHC^+^ fiber diameters than the other groups; however, this difference reached statistical significance only when group 1 dMyHC^+^ fiber diameters were compared to those of group 2 (Fig. 3C).

**Fig. 3.**
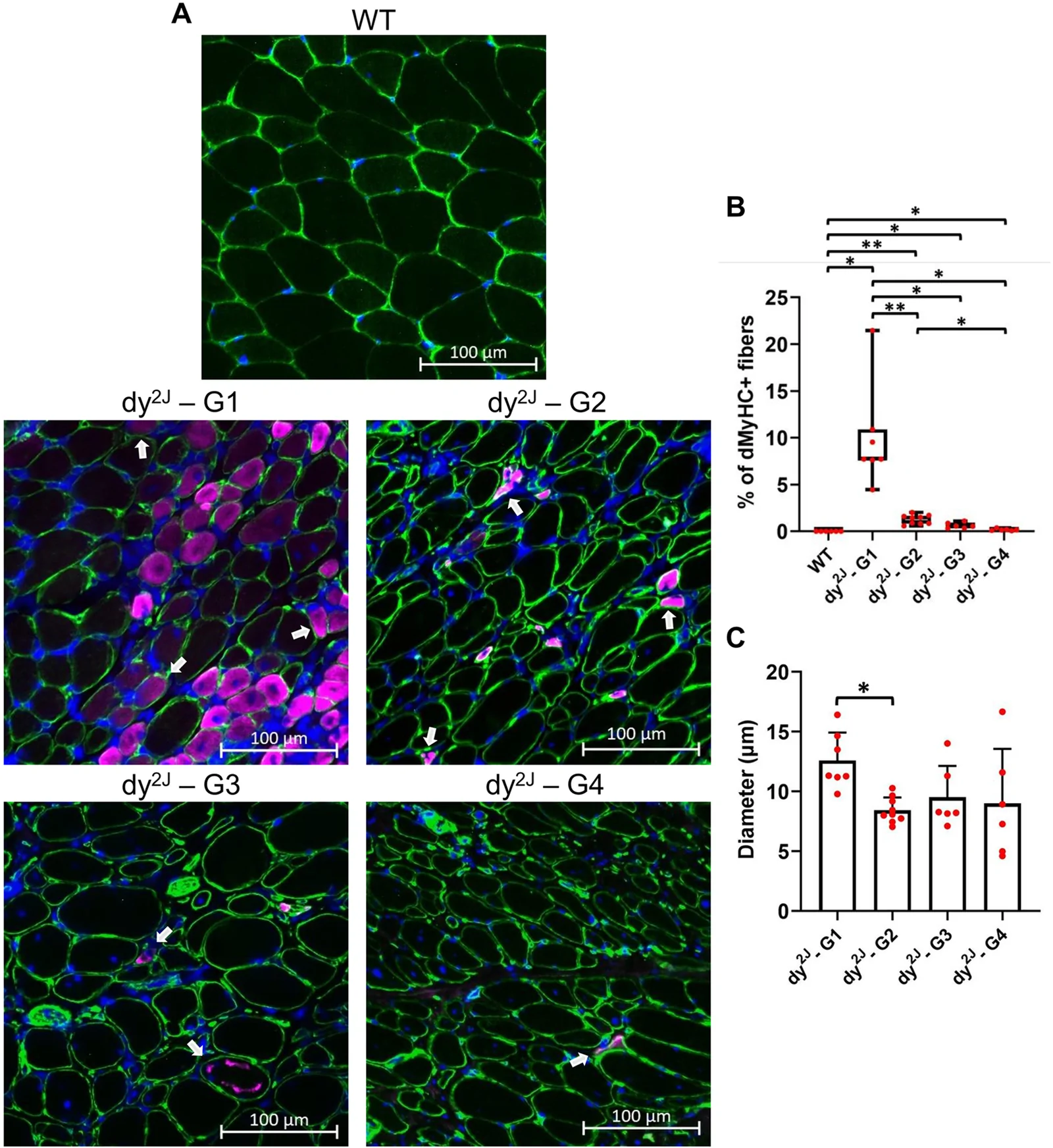
**Newly regenerated fibers in dy^2J^ groups.** (A) Immunofluorescence staining of laminin (green) and developmental myosin heavy chain (dMyHC; magenta) across the WT group and dy^2J^ age groups. Blue indicates nuclei. White arrows indicate dMyHC^+^ fibers. (B) Percentage of dMyHC^+^ fibers in relation to total fiber in the WT group and in the four dy^2J^ age groups. Box-and-whisker plots show median (center line), 25th-75th percentiles (box limits) and mimumin-to-maximum range (whiskers). (C) Muscle dMyHC^+^ fiber diameter across dy^2J^ age groups. Bars represent mean±s.d. Individual data points represent biological replicates (individual animals). Group sizes: WT, six animals; dy^2J^-G1, seven animals; dy^2J^-G2, nine animals; dy^2J^-G3, six animals; dy^2J^-G4, six animals. Statistical analysis was performed using Kruskal–Wallis for percentage of dMyHC^+^ fibers (*P*<0.001), with DSCF for multiple comparisons. One-way ANOVA was performed for dMyHC^+^ fiber diameter (*P*=0.024), with Games–Howell post hoc for multiple comparisons. *P<0.05 and **P<0.01.

### Fiber size variability is high and muscle fiber diameter progressively declines with disease progression

Histopathological analysis is fundamental for understanding the impact of the dy^2J^ mutation on skeletal muscle. Assessing mice at different stages of dystrophy progression further clarifies the consequence of progressive regenerative failure. Accordingly, Hematoxylin and Eosin (H&E) staining was performed on gastrocnemius sections from dy^2J^ mice across the four dy^2J^ age groups and in the WT group. All dy^2J^ age groups displayed hallmark features of dystrophic muscle, including centralized nuclei, fibrosis infiltration and fiber size variability (Fig. 4A). Furthermore, the coefficient of variation of muscle fiber diameters was greater than 250 in all groups, exceeding the normal threshold for fiber size variability. The mean fiber diameter was significantly lower in all dy^2J^ groups than in the WT group (Fig. 4B). This progressive decline was particularly evident in the most advanced stage (group 4), which exhibited significantly smaller fibers than did groups 1 and 3 (Fig. 4B). These changes were reflected in the fiber size distribution; although WT fibers were predominantly between 40 and 60 μm in diameter, fiber diameters of dy^2J^ groups 1-3 centered around 20-40 μm, with group 4 showing a further shift toward the 0-20 μm interval (Fig. 4C). Consistent with this generalized atrophy, the number of fibers per area was significantly higher in dystrophic groups 2, 3 and 4 compared to WT, with group 4 displaying the highest fiber density (Fig. 4D).

**Fig. 4.**
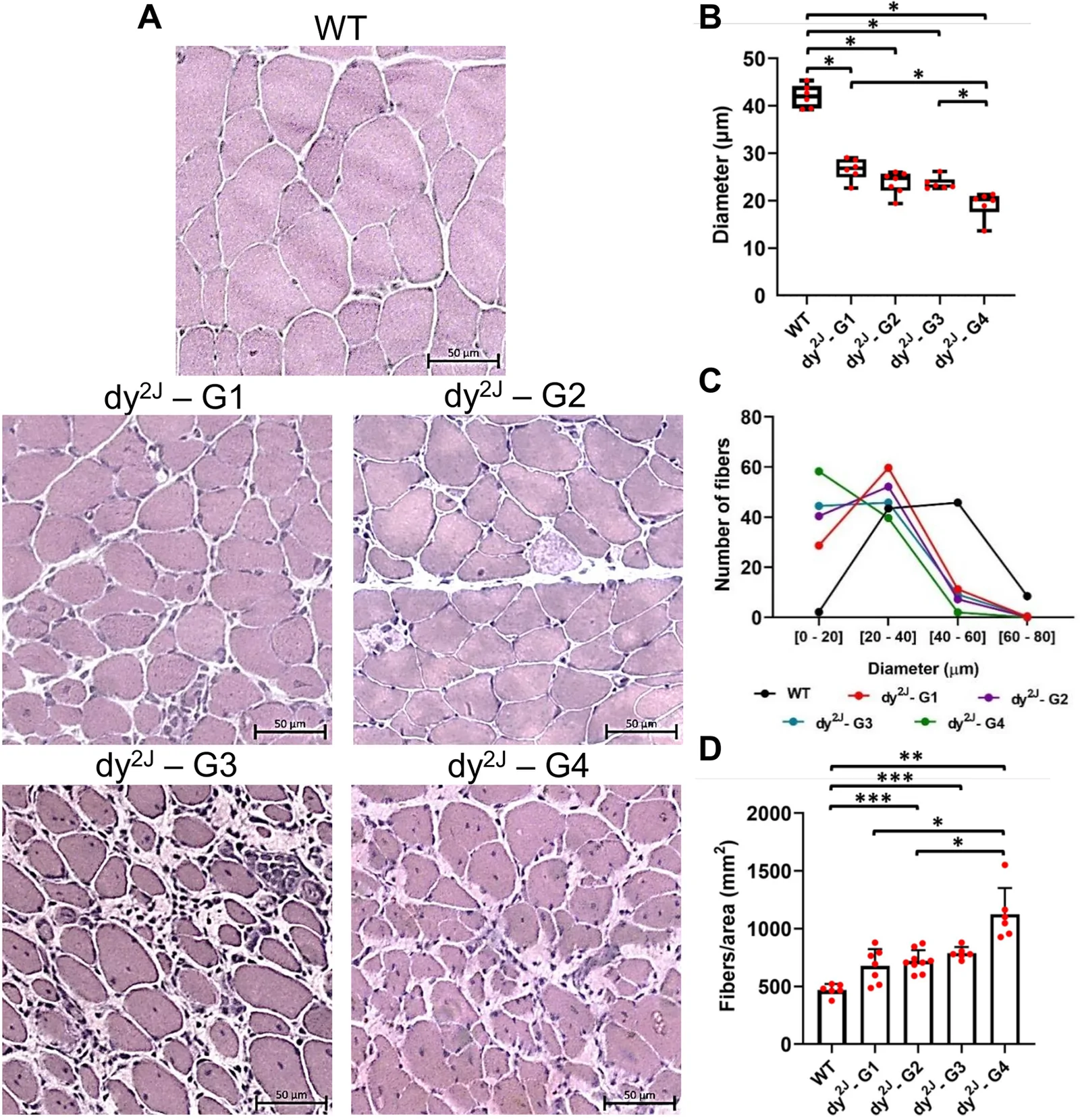
**Histopathological analyses.** (A) Representative Hematoxylin and Eosin staining images of skeletal muscle from the WT group and the different dy^2J^ age groups. (B) Muscle fiber diameter across the WT and dy^2J^ age groups. Box-and-whisker plots show median (center line), 25th-75th percentiles (box limits) and minimum-to-maximum range (whiskers). A total of 100 fibers were measured per animal. (C) Distribution of muscle fiber diameters. (D) Number of fibers per area (mm^2^) in the WT group and the different dy^2J^ age groups. Bars represent mean±s.d. Individual data points represent biological replicates (individual animals). Group sizes: WT, six animals; dy^2J^-G1, six animals; dy^2J^-G2, nine animals; dy^2J^-G3, six animals; dy^2J^-G4, six animals. Statistical analysis was performed using Kruskal–Wallis for fiber diameter (*P*<0.001), with DSCF for multiple comparisons. One-way ANOVA was used for fibers/area (*P*<0.001), with Games–Howell post hoc for multiple comparisons. *P<0.05, **P<0.01 and ***P<0.001.

### Collagen deposition is higher in dy^2J^ groups than in the WT group

Replacement of contractile muscle tissue by fibrotic deposition is a hallmark of muscular dystrophies. This process not only impairs muscle function through fiber loss but also disrupts the ECM architecture and intercellular signaling. Therefore, quantifying fibrosis is essential to monitor disease progression. To this end, Picrosirius Red staining (Fig. 5A) was performed to assess collagen deposition. Consistent with the dystrophic phenotype, all dy^2J^ groups exhibited a significant increase in collagen area fraction compared to that in the WT group (Fig. 5B), indicating early-onset fibrosis in this model. Notably, group 3 showed significantly greater collagen deposition than group 2, suggesting a peak in fibrotic accumulation as the disease advances.

**Fig. 5.**
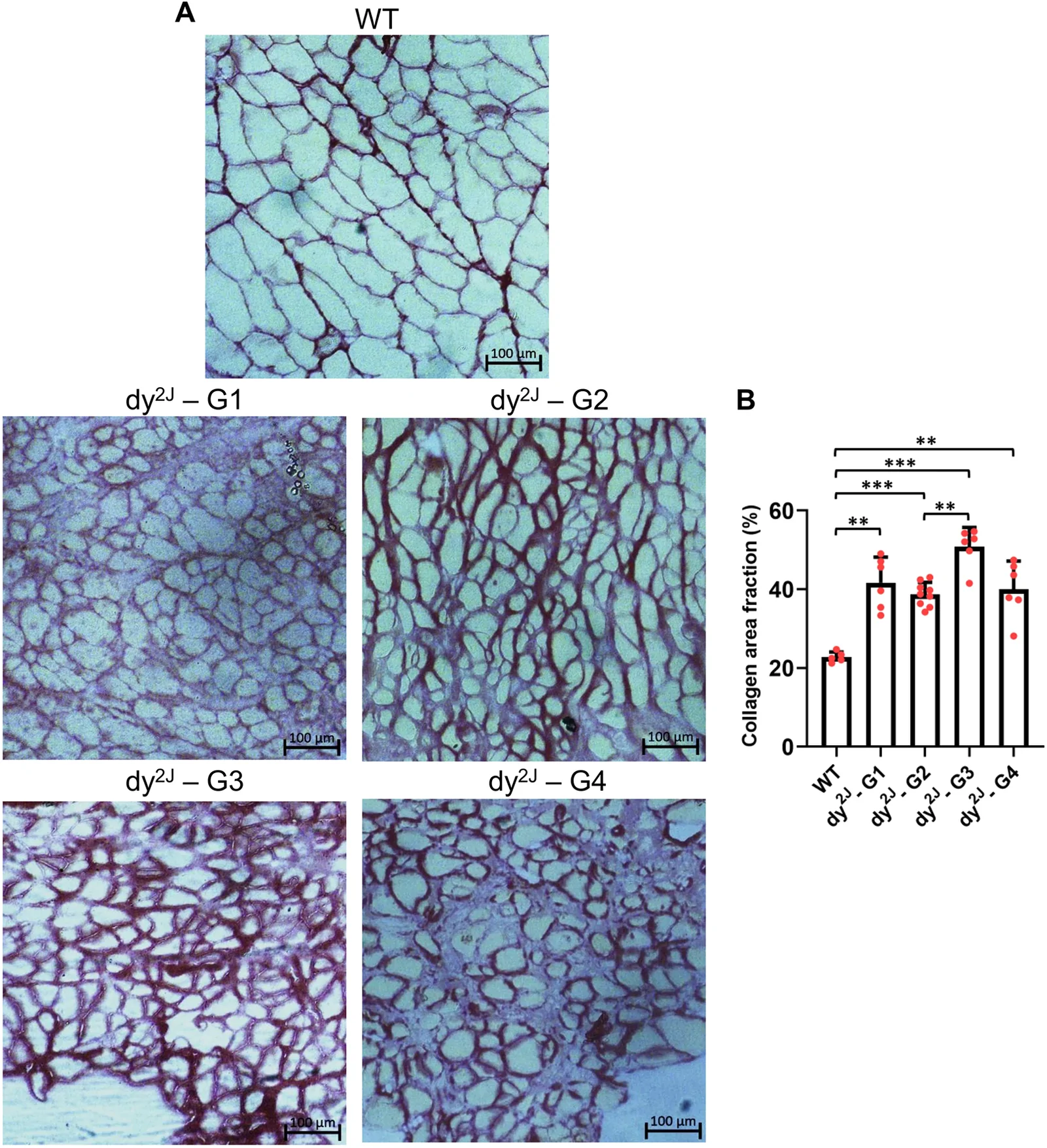
**Histological assessment of collagen deposition.** (A) Representative Picrosirius Red staining images of skeletal muscle from the WT group and the different dy^2J^ age groups. (B) Collagen area fraction across the WT and dy^2J^ age groups. Bars represent mean±s.d., with individual points representing biological replicates (individual animals). Group sizes: WT, five animals; dy^2J^-G1, six animals; dy^2J^-G2, nine animals; dy^2J^-G3, six animals; dy^2J^-G4, six animals. Statistical analysis was performed using one-way ANOVA (*P*<0.001), with Games–Howell post hoc for multiple comparisons. **P<0.01 and ***P<0.001.

## DISCUSSION

In muscular dystrophies, chronic muscle degeneration provides a persistent regenerative stimulus. However, muscle is unable to fully compensate for the ongoing damage, resulting in progressive muscle loss (Bencze, 2022). Consequently, the ability of SCs to sustain muscle regeneration throughout all stages of disease has been widely questioned, as the muscle stem cell (SC) pool may undergo replicative aging, senescence or eventual depletion. Furthermore, it has been suggested that the degenerative microenvironment hinders the ability of SCs to differentiate effectively (Sousa-Victor et al., 2022; Wallace and McNally, 2009).

Nevertheless, recent evidence, including findings from our group, indicate that the SC pool and its proliferative capacity are preserved, regardless of the severity of degeneration (Kottlors and Kirschner, 2010; Ribeiro et al., 2019). Our results here corroborate this observation: continuous SC activation did not necessarily lead to depletion, as dy^2J^ mice exhibited significantly higher numbers of PAX7^+^ SCs than did WT mice across all age groups. SCs retained activation potential, particularly at early stages (group 1), as evidenced by the presence of PAX7^+^/KI67^+^ cells. However, the marked upregulation of *Myog* suggests a premature shift toward terminal differentiation.

The increased *Myog* expression, together with the relatively low proportion of PAX7^+^/KI67^+^ cells in advanced stages of disease progression (groups 2-4), suggests a reduction in SC proliferative capacity rather than a failure in myogenic commitment. This pattern indicates that dy^2J^ SCs enter differentiation without sufficient prior expansion. This is consistent with recent findings from McGowan et al. (2025), showing that loss-of-function mutations in *Lama*2 intrinsically slow cell-cycle progression and hinder SC expansion in mice and humans. Notably, despite these proliferative defects, *Lama2*-deficient cells retain their full capacity to differentiate and fuse into multinucleated myotubes, as evidenced by a normal fusion index and preserved differentiation markers *ex vivo* (McGowan et al., 2025). Therefore, the elevated *Myog* levels in our model likely reflect a conserved, yet inefficient, regenerative response in which differentiation proceeds despite the failure to establish an adequate progenitor pool.

This impaired muscle regeneration is evidenced by the reduction in percentage and mean diameter of dMyHC^+^ regenerated fibers in dy^2J^ muscle, suggesting that the capacity of SCs to complete differentiation is worsened in the later stages of the disease. Additionally, the finding that the mean diameter of total fibers is significantly reduced in dy^2J^ mice compared to that in WT mice across all age groups reinforces the presence of defective muscle regeneration. Accordingly, dy^2J^ SCs appear unable to generate fibers of normal caliber, even at early stages. As the pathology progresses, repeated cycles of degeneration, coupled with inefficient regeneration, result in the cumulative replacement of the muscle by progressively smaller fibers. This generalized atrophy may be further exacerbated by other factors, such as enhanced autophagic and apoptotic signaling, as laminin-α2 is essential for maintaining cellular homeostasis and regulating these processes (Carmignac et al., 2011a,b; Körner et al., 2014; Mastrapasqua et al., 2023).

The degenerative microenvironment may also impact regeneration in the dy^2J^ model, with fibrosis representing a particularly relevant contributing factor. Fibrosis is defined by the excessive accumulation of ECM proteins (Mann et al., 2011). ECM components directly interact with cells via specific receptors, thereby modulating key biological processes such as cell fate determination, proliferation, migration and adhesion (Loreti and Sacco, 2022). In this context, TGF-β1 plays an important role, stimulating the expression of ECM proteins, such as collagen, driving fibrotic remodeling (Delaney et al., 2017). In our study, we observed elevated *Tgfb* expression and increased collagen deposition in dy^2J^ muscles compared to that in WT muscles, indicating dysregulation of fibrotic pathways. Pharmacological strategies targeting these mechanisms have demonstrated efficacy in preclinical models by reducing collagen accumulation and partially restoring muscle architecture (Dadush et al., 2010; Elbaz et al., 2012; Nevo et al., 2010). Beyond fibrosis, chronic exposure to inflammation and repeated cycles of degeneration and regeneration represent additional extrinsic factors that further compromise the dystrophic microenvironment. Sustained inflammatory signaling has been shown to induce telomere shortening in SCs (Tichy et al., 2021), while successive rounds of tissue repair can lead to alteration in their epigenetic landscape (Falick Michaeli et al., 2022). These persistent ‘molecular scars’ likely contribute to the progressive decline in regenerative capacity, creating a deleterious synergy between intrinsic laminin-α2 deficiency and a hostile niche that cumulatively drives the severe muscle wasting observed in dy^2J^ mice (McGowan et al., 2025).

Understanding both intrinsic and extrinsic barriers to muscle regeneration is essential for the development of effective therapeutic strategies. Current interventions aim not only to restore myogenic potential but also to address the underlying protein deficiency. Direct replacement of the *LAMA2* gene or delivery of laminin-211 remain the principal therapeutic goals for laminin-α2-related muscular dystrophy. In the dy^2J^ mouse model, which displays laminin deficiency resulting from a point mutation in *Lama2* gene, correction is comparatively less complex than in models carrying large deletions and has been successfully achieved. This approach has led to significant phenotypic improvement (Kemaladewi et al., 2019a).

Additionally, upregulation of laminin-111 has emerged as a powerful disease-modifying strategy. Laminin-111 can functionally compensate for the absence of laminin-α2 chain, partially restoring basement membrane integrity and improving the mechanical stability of the sarcolemma. This approach has been successfully demonstrated in dy^2J^ mice through both transgenic overexpression of laminin-α1 (Gawlik et al., 2018) and CRISPR/dCas9-mediated activation of laminin-α1 expression (Kemaladewi et al., 2019b). In other LAMA2 MD mouse models, laminin-α1 overexpression has also shown significant therapeutic benefits. In dy^3K^ mice, transgenic expression of laminin-α1 markedly ameliorates muscle pathology, improves overall health and extends survival to ∼1.5-2 years of age (Gawlik and Durbeej, 2010; Gawlik et al., 2004). Furthermore, systemic administration of laminin-111 protein improves muscle viability and disease outcomes in dy^W^ mice, both when treatment is initiated early in life (Rooney et al., 2012) and after disease onset (Barraza-Flores et al., 2020b). Transgenic overexpression is technically challenging in patients owing to the large size of the *LAMA2* gene (Barraza-Flores et al., 2020a; Yurchenco et al., 2018). To overcome these limitations, alternative strategies employing laminin-linker proteins have shown considerable promise in re-establishing basement membrane integrity and improving muscle function in severe models. Notably, in the dy^2J^ mouse model, transgenic muscle-specific expression of a laminin–nidogen chimeric protein (αLNNd) markedly ameliorated the dystrophic phenotype. This therapeutic effect was achieved through restoration of the laminin polymerization function that is compromised by the dy^2J^ mutation, thereby improving basement membrane organization and muscle pathology (McKee et al., 2017).

A similar approach was used in the dy^W^ model, which is characterized by complete absence of laminin-α2 and a compensatory increase in laminin-411 expression. However, laminin-α4 does not bind efficiently to α-dystroglycan and lacks self-polymerization capacity (Yurchenco et al., 2018). To overcome this limitation, Reinhard et al. (2017) induced transgenic, muscle-specific expression of αLNNd and mini-agrin, which binds to the coiled-coil domain of laminins at one end and to α-dystoglican at the other. This combined expression improved muscle pathology in dy^W^ mice (Reinhard et al., 2017).

In conclusion, our results demonstrate that the progressive impairment of muscle regeneration in the dy^2J^ model is not caused by depletion of the SC pool. Rather, regeneration appears to fail at the late stages of SC differentiation. This impairment may arise as a direct consequence of the primary laminin-α2 deficiency, and/or from the compromised dystrophic microenvironment. Further investigation is required to better elucidate the dynamic interplay, and advancing our understanding of these mechanism will be essential for the development of effective treatment strategies.

## MATERIALS AND METHODS

### Animals

This study was approved by the Animal Ethics Committee of the Institute of Biosciences, University of São Paulo (CEUA protocol no. 413/2023) and conducted in accordance with institutional guidelines. dy^2J^ and C57BL/6 (WT) mice of both sexes were used, and analyses were performed *ex vivo* on gastrocnemius muscle tissue. A total of 33 dy^2J^ mice were analyzed and distributed into four age groups corresponding to developmental stages: adolescence (1-2 months old), young adult (2-8 months old), middle age (8-15 months old) and old age (>15 months old) (Arellano et al., 2024). For comparison, seven WT mice at the young-adult stage were included. At least five animals per group were analyzed in each experimental group. All animals were bred under controlled temperature and light conditions in the animal facility of the Human Genome and Stem Cell Research Center (HUG-CELL). Animals were genotyped for the correct genetic mutation of the model (Vilquin et al., 2000).

### Muscle acquisition

The animals were anesthetized and euthanized, in accordance with the guidelines of the National Council for the Control of Animal Experimentation (CONCEA). Then, the gastrocnemius and soleus muscles were collected. One pair of these muscles was embedded in TissueTek O.C.T. (Qiagen) on cork blocks coated with talc for cryoprotection. The other muscle fragments were macerated and stored frozen in cryotubes for subsequent RNA extraction.

### RNA extraction and cDNA synthesis

RNA extraction was performed using TRIzol reagent (Invitrogen, Thermo Fisher Scientific), following the manufacturer's instructions. After extraction, the RNA was diluted in nuclease-free water (Ambion, Thermo Fisher Scientific). RNA integrity was then assessed using a NanoDrop 1000™ spectrophotometer (Thermo Fisher Scientific). For cDNA synthesis, 1 μg total RNA was combined with 4 μl SuperScript™ ViLO Master Mix (Invitrogen, Thermo Fisher Scientific) in a final volume of 20 μl. The reaction mixture was incubated in a thermocycler at 25°C for 10 min, 42°C for 60 min and 85°C for 5 min.

### SC identification and analysis

For the identification of quiescent and activated SCs, concomitant immunofluorescent staining of PAX7 and KI67 was performed. The slides were first incubated with 4% paraformaldehyde for 10 min, followed by two 5-min washes with PBS with 0.1% Triton X-100 (PBST). Antigen retrieval was then performed using citrate buffer in a pressure cooker for 5 min, after which the slides were washed again twice for 5 min with PBST. Next, the slides were incubated in blocking solution for 1 h at room temperature, followed by three 5-min washes with PBST.

Slides were then incubated overnight at 4°C with primary antibodies anti-PAX7 (1:10; Developmental Studies Hybridoma Bank) and anti-KI67 (1:200; Abcam, ab15580). The following day, they were washed three times with PBST (5 min each) and incubated for 1 h at room temperature with secondary antibodies Cy3-labeled sheep anti-mouse IgG (1:100; Sigma-Aldrich, C2181) and FITC-labeled goat anti-rabbit IgG (1:200; Invitrogen, A11008). Finally, the slides were washed three more times with PBST and mounted with Vectashield (Vector Laboratories) supplemented with DAPI (Boehringer Mannheim, Roche).

Images were acquired at 400× magnification using a Zeiss confocal microscope. Using ImageJ software, the numbers of quiescent and activated SCs, as well as the numbers of muscle fibers, were quantified. At least 1000 fibers were counted per animal.

### Gene expression analyses (real-time PCR)

Gene expression analysis of *Pax7*, *Myf5*, *Myod*, *Myog* and *Tgfb* was performed in different age groups of dy^2J^ mice and in the juvenile WT mice, which were used for expression normalization. For real-time PCR, each sample was run in triplicate on a 96-well plate. Each well contained 7.4 μl RNase-free water, 0.3 μl of each primer for the target gene, 10 μl PowerUP SYBR Green Master (Applied Biosystems, Thermo Fisher Scientific) and 2 μl cDNA (diluted 1:10 in RNase-free water), for a total volume of 20 μl. The sequences of the primers used in the reactions are listed in Table S1. The plate was placed in an Applied Biosystems QuantStudio5 PCR System and run using the following cycling conditions: 95°C for 10 min, followed by 40 cycles of 95°C for 15 s and 60°C for 1 min. Relative expression levels were determined using the comparative 2^−ΔΔCt^ method, with target gene expression normalized to the endogenous reference gene *Tbp* and calibrated against the WT control group.

### Histological sectioning

The frozen muscles embedded in TissueTek O.C.T. were sectioned using a cryostat (Microm HM 505E, Thermo Fisher Scientific) at a temperature of −27°C. Sections were cut at a thickness of 7 μm and mounted transversely on slides. The slides were stored at −70°C until use.

### Newly regenerated fiber identification and analysis

The analysis of regenerating muscle fibers was performed by immunofluorescence against dMyHC, a protein expressed during the early stages of muscle formation and in newly regenerated fibers (Schiaffino et al., 2015). The slides were covered with PBS and incubated overnight with primary antibodies anti-dMyHC (1:30; Vector Laboratories, VP-M664) and anti-laminin (1:200; Dako, Z0097). After incubation, the slides were washed with PBS and then incubated for 1 h with secondary antibodies Cy3-labeled sheep anti-mouse IgG (1:100; Sigma-Aldrich, C2181) and FITC-labeled goat anti-rabbit IgG (1:200; Invitrogen, A11008). The slides were then washed again with PBS and mounted with Vectashield containing DAPI, a nuclear stain.

Image acquisition was performed using a fluorescence microscope (Zeiss), capturing the entire histological section at 100× magnification. An average of 15 images per slide was taken and analyzed using ImageJ software. Newly formed fibers were quantified relative to the total number of fibers. In addition, the minimum diameter of the newly regenerated fibers was measured.

### Histopathological analysis

For histopathological analysis, H&E staining was performed. The slides were stained with Hematoxylin for 10 min and then washed three times with distilled water. Next, they were stained with Eosin for 3 min and rinsed to remove excess dye. After staining, the sections were fixed with acetic acid, dehydrated through increasing concentrations of alcohol and xylene, and mounted with Canada balsam. For analysis, the sections were photographed using a light microscope (Zeiss) at 200× magnification. Finally, Feret’s minimum diameter of 100 muscle fibers per animal was measured using ImageJ software. The fiber size variability coefficient was calculated (standard deviation×1000/mean fiber diameter), and a result greater than 250 was considered pathogenic (Dubowitz et al., 2022).

### Collagen deposition analysis

For collagen deposition analysis, Picrosirius Red staining was performed. The slides were stained with Picrosirius Red for 50 min. After staining, the sections were washed with acetic acid, dehydrated through increasing concentrations of alcohol and xylene, and mounted with Canada balsam. For analysis, the sections were photographed using a light microscope (Zeiss) at 100× magnification. ImageJ automatic threshold was used to determine the collagen area fraction.

### Statistical analysis

Statistical analyses were performed in Jamovi, with a significance level set at α=0.05 for all tests. Data were first assessed for normality using the Shapiro–Wilk test for each group. If all groups showed normal distribution, homogeneity of variances was tested using Levene's test. When both assumptions were met, a one-way ANOVA was applied, followed by Tukey's post hoc test. If variances were unequal, Games–Howell post hoc was used instead. If at least one group violated the normality assumption, the Kruskal–Wallis test was applied. When significant differences were found, pairwise comparisons were conducted using the Dwass–Steel–Critchlow–Fligner method. A post hoc power analysis was performed for variables that met normality assumptions and were analyzed by one-way ANOVA, in order to assess sample size adequacy. Effect sizes (η^2^) obtained in Jamovi were converted to Cohen's *f*, and statistical power was calculated using the non-central *F* distribution (α=0.05). The primary outcome (PAX7^+^ cells/fiber; η^2^=0.543, *f*=1.09) yielded an observed power of 0.999. Most secondary outcomes also exceeded the conventional 0.80 threshold. The analysis of dMyHC^+^ fiber diameter yielded a power of 0.759, approaching the conventional threshold, whereas *Pax7* and *Myf5* mRNA expression showed lower power (0.43), consistent with their small effect sizes (η^2^≈0.17) and lack of statistically significant differences. Group sizes (six to nine animals) were determined by the study design, which emphasized comparisons across developmental stages rather than maximizing sample size within a single age group.

## Supplementary Material

10.1242/dmm.052782_sup1 Supplementary information
