# Supplementary material for: Preserved satellite cell pool in a laminin-α2-deficient model of congenital muscular dystrophy
Source: Dis Model Mech. 2026 Sep 30;19(9):dmm052782. doi: 10.1242/dmm.052782 (PMC13670365; doi:10.1242/dmm.052782)
Supplement: Supplementary file 1 [file dmm-19-052782-s1.pdf]

Table S1. Forward and reverse primers used for analysis of the relative expression of genes *Tbp*, *Pax7*, *Myf5*, *Myod*, *Myog* and *Tgfb*

Gene	Forward primer	Reverse primer
Tbp	TGCACAGGAGCCAAGAGTGAA	CACATCACAGCTCCCCACCA
Pax7	GAGTTCGATTAGCCGAGTGC	GTGTTTGGCTTTCTTCTCGC
Myf5	GAAGGTCAACCAAGCTTTCG	GCTCTCAATGTAGCGGATGG
Myod	TACAGTGGCGACTCAGATGC	TAGTAGGCGGTGTCGTAGCC
Myog	CAGTACATTGAGCGCCTACAG	GGACCGAACTCCAGTGCAT
Tgfb	CCCCACTGATACGCCTGAGT	AGCCCTGTATTCCGTCTCCTT
